# Malignant transformation of pleomorphic xanthoastrocytoma and differential diagnosis: case report

**DOI:** 10.1186/s12883-020-1601-2

**Published:** 2020-01-15

**Authors:** Noriyuki Watanabe, Eiichi Ishikawa, Hidehiro Kohzuki, Noriaki Sakamoto, Alexander Zaboronok, Masahide Matsuda, Makoto Shibuya, Akira Matsumura

**Affiliations:** 1 Departments of Neurosurgery, Faculty of Medicine, University of Tsukuba, 1-1-1 Tennodai, Tsukuba, Ibaraki Japan; 2 Departments of Diagnostic Pathology, Faculty of Medicine, University of Tsukuba, Tsukuba, Ibaraki Japan; 3 Central Laboratory, Hachioji Medical Center, Tokyo Medical University, Tokyo, Japan

**Keywords:** Pleomorphic xanthoastrocytoma, Malignant transformation, Glioblastoma, Tumor recurrence, Differential diagnosis

## Abstract

**Background:**

Pleomorphic xanthoastrocytoma (PXA) is a rare astrocytic glioma, characterized by large pleomorphic and frequently multinucleated cells, spindle and lipidized cells, a dense pericellular reticulin network, and numerous eosinophilic granular bodies according to the grade II glial tumor standards of the World Health Organization’s (WHO) 2016 guidelines. PXA rarely transforms into anaplastic PXA or glioblastoma (GBM) and anaplastic PXA, classified as WHO grade III, has a more aggressive clinical behavior with poorer prognosis than PXA.

**Case presentation:**

Here we describe an unusual case of PXA in a 19-year-old woman, first admitted with headache and a mass in the left temporal lobe in 2005 that was removed. Twelve years later, she returned with left temporal headache, diplopia and tinnitus. A local tumor recurrence was found, and a second resection was performed. The specimen showed highly malignant findings, such as necrosis, microvascular proliferation, and multiple mitoses. The integrated diagnosis was made as high grade glioma, probably derived from PXA. Immunohistochemical (IHC) stains were positive for oligo2, and approximately 21% positive for Ki-67, while negative for CD34, IDH1 R132H. INI1 and ATRX were retained. As the histological classification was glioblastoma, the patient received GBM-appropriate chemotherapy and radiation therapy and outpatient follow-ups have demonstrated no obvious symptoms for 1 year after surgery. Additional molecular analyses found BRAF V600E mutations in both resections, supporting the idea that the recurrent tumor had derived from PXA.

**Conclusions:**

This case highlights the complexities of differential diagnosis based on the World Health Organization’s 2016 guidelines. More integrated criteria to differentiate anaplastic PXA from GBM and epithelioid GBM, combined with genetic screening results, might be needed.

## Background

Pleomorphic xanthoastrocytoma (PXA) is a rare astrocytic glioma, characterized by large pleomorphic and frequently multinucleated cells, spindle and lipidized cells, a dense pericellular reticulin network, and numerous eosinophilic granular bodies (EGBs) according to the grade II glial tumor standards of the World Health Organization’s (WHO) 2016 guidelines [1]. PXA rarely transforms into anaplastic pleomorphic xanthoastrocytoma (APXA) or glioblastoma (GBM). APXA, classified as WHO grade III, has a more aggressive clinical behavior with poorer prognosis than PXA [2, 3]. Histologically, APXA is defined when, in addition to PXA, mitoses number 5 or more per high-power field while GBM displays nuclear atypia, cellular pleomorphism, mitotic activity, a diffuse growth pattern, microvascular proliferation and/or necrosis [1]. Thus, these tumors have similar histological findings which may cause clinical confusion [4, 5]. BRAFV600 E mutations are seen in 7.7~9.1% of GBM, 63~75% of PXA and 47.4~57% of APXA cases [1, 3, 6, 7].

Although the WHO 2016 classification incorporated molecular testing, diagnoses of anaplastic PXA and GBM are still based on histopathological findings. Here, we present a case of such a complex differential diagnosis of GBM, solely by histology, after taking the clinical course and molecular findings into account.

## Case presentation

**Fig. 1:**
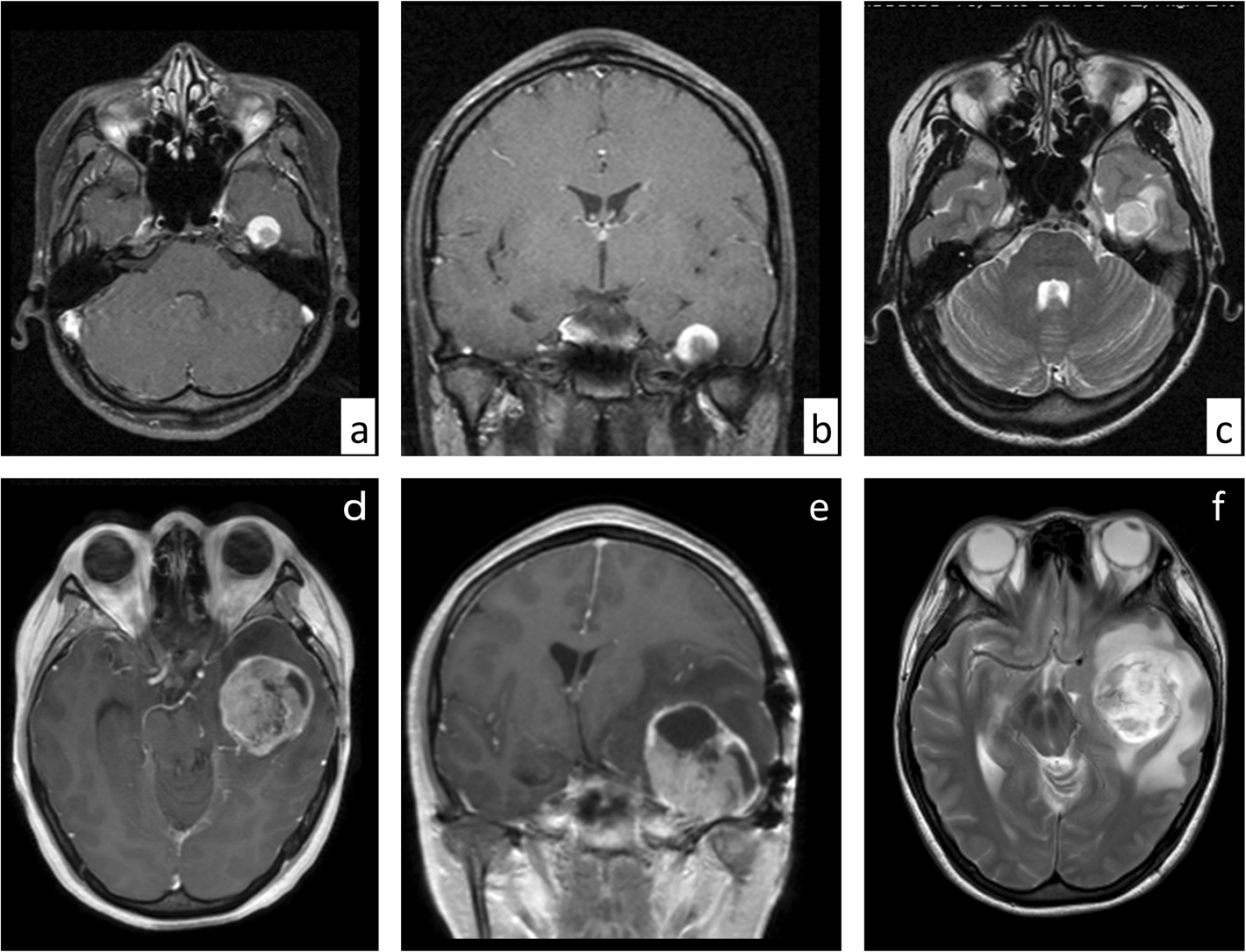
Neuroimaging along the course of the disease. T1-weighted MR images with gadolinium contrast (**a**, **b**) revealing a well-defined, heterogeneously enhanced tumor in the left temporal lobe, approximately 10 mm in size, with high intensity on T2-weighted imaging (**c**). Axial(**d**) and coronal(**e**) MR images with gadolinium contrast and axial T2-weighted images (**f**) showing local recurrence in the surgical cavity 12 years after the initial treatment with a size of approximately 42 × 45 × 47 mm

A 19-year-old Japanese woman presented with a left temporal headache. Magnetic resonance imaging (MRI) of the brain revealed a well-defined, heterogeneously enhanced tumor in the left temporal lobe, approximately 10 mm in size, showing high-intensity on T2-weighted images (Fig. 1a, b, c). The patient underwent a left frontal-temporal craniotomy and total resection. These tumor cells had less than 1 mitosis per 10 high-power fields. The primary histopathological diagnosis at that time was low-grade glioma. The patient was discharged after treatment and followed up without any additional treatment. No clear local recurrence was detected during 3 years of follow-up and the patient subsequently elected to stop further medical examinations.

**Fig. 2:**
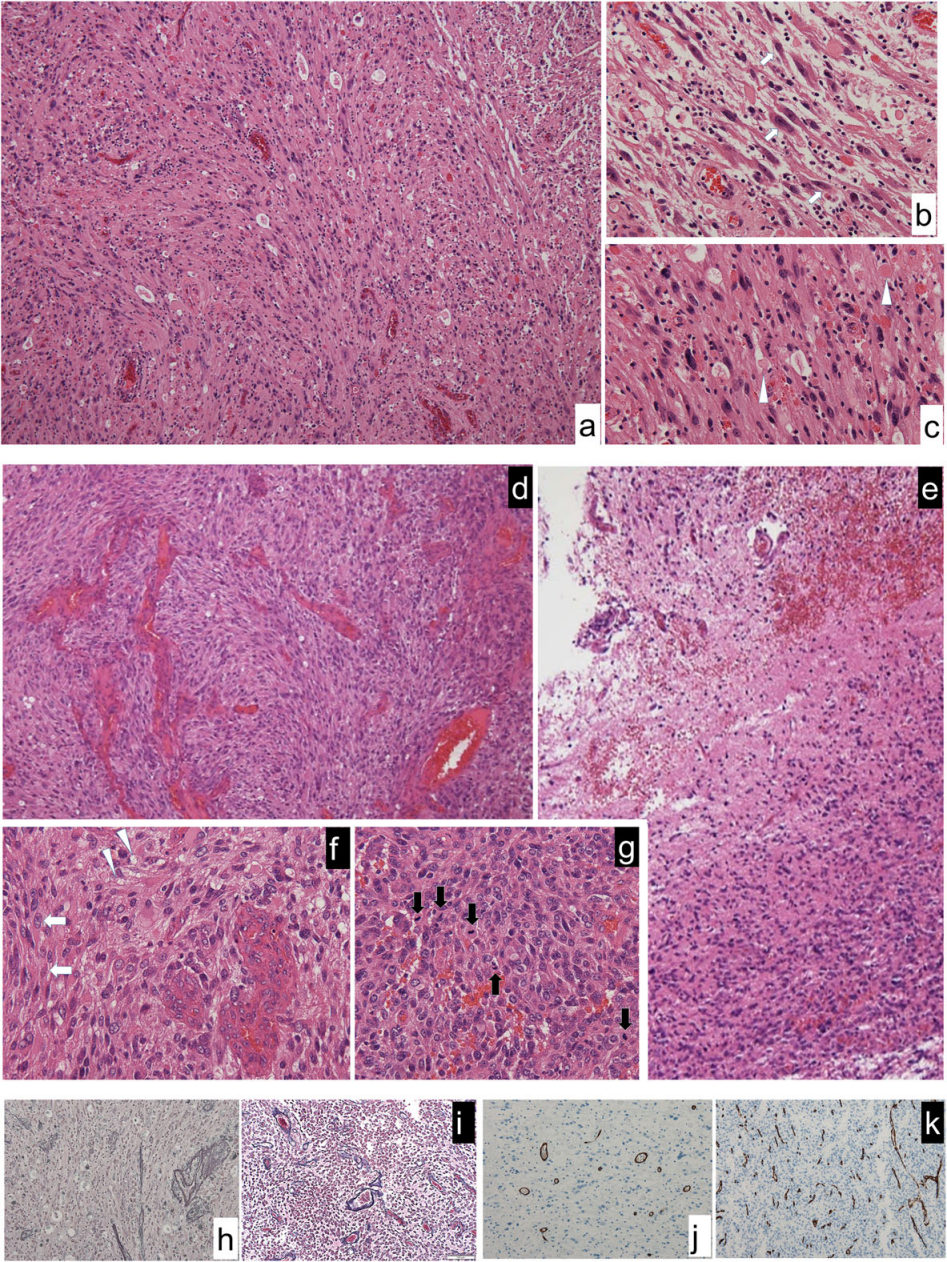
Microscopic findings of tumors in the initial and recurrent specimens. Spindle cells with nuclear atypia (arrows) with diffusely infiltrating lymphocytes, and eosinophilic granular bodies (arrow heads) as seen in the first resection. (hematoxylin and eosin (H&E) stain, **a** × 200, **b** and **c** × 400). In the recurrent specimen, broad necrosis and infiltrating tumor cells can be observed, and massively increased cellularity and prominent atypia are present along the relatively well-defined border line (**d** and **e**, H&E stain, × 200). With microvascular proliferation, only a small proportion of spindle shape cells (arrows) and xantic cells (arrow heads) can be observed, implying similarity to the previous resection (**f**, H&E stain, × 400). Multiple mitoses are visible (black arrows), indicating malignancy (**g**, H&E stain, × 400). No reticulin fibers were observed in the first (**h**) and second (**i**) specimens (reticulin stain, × 200). On CD34 staining, tumor cells were not stained in the first (**j**) and second (**k**) specimens (CD34 stain, × 200)

However, 12 years after the initial treatment, the patient returned with a left temporal headache, diplopia and tinnitus. MRI showed a local recurrence of the tumor around the surgical cavity with prominent perifocal edema (Fig. 1d, e, f) and a second resection was performed. Intraoperatively, the tumor was prominently hypervascular, with many feeding arteries from the dura, and a part of the tumor was weakly positive for 5-aminolevuinic acid-based photodynamic diagnosis (PDD). Hematoxylin and eosin (H&E) staining showed an astrocytic tumor lesion with necrosis, microvascular proliferation, invasion and multiple mitoses (5 mitotic counts per 10 high-power fields). No EGBs were observed. (Fig. 2d-g). Immunohistochemical (IHC) stains were positive for oligo2, and tumor cells were retained for INI1 and ATRX staining. Approximately 21% of these cells were positive for Ki-67 but negative for IDH1-R132H. Furthermore, the specimen from the initial surgery was revisited and the diagnosis was changed to PXA of WHO grade II based on histology showing spindle shaped cells, pleomorphic nucleated cells and EGBs (Fig. 2a, b, c). Reticulin fibers were not stained and CD34 staining was negative in both tumor resections (Fig. 2h-k). As recurrence happened after the PXA resection, APXA was expected due to poorer histological findings; however, the tumor had no characteristic feature of PXA such as EGB, leading to a diagnosis as GBM by histological classification because of nuclear atypia, mitotic activity, a diffuse growth pattern, microvascular proliferation and necrosis (Table 1). The patient received chemotherapy (temozolomide) and conformal radiation therapy accordingly and outpatient follow-ups have detected no obvious symptoms for over 1 year after surgery.

**Table 1:** Histological and immunohistological comparison among disease entities (based on Giannini et al. 2016 [1])

	GBM (wild type)	PXA	APXA	Present case (1st resection)	Present case (2nd resection)
Mitosis (per 10 HPF)	+	< 5	≧5	< 1	5
Necrosis	-~+	Rare	-~+	–	+
MVP	-~+	NA	uncommon	–	+
Pleomorphism	-~+	+	+	+	+
IDH mutation	–	–	–	–	–
ATRX mutation	–	NA	NA	–	–
GFAP	+	+	+	+	+
p53	-~+	Variable	NA	- (< 10%)	+ (15%)
MIB1	15–20% or more	<2~5%	NA	1%	21%

*GBM* glioblastoma, *PXA* pleomorphic xanthoastrocytoma, *APXA* anaplastic PXA, *HPF* high-power field, *NA* not available, *MVP* microvascular proliferation

Additionally, specimens of each resection were sent to the National Cancer Center Research Institute and MLPA (Multiplex Ligation-dependent Probe Amplification) was performed for 1p, 19q, CDKN2A, IDH1 R132H (c.395G > A), R132C (c.394C > T), IDH2 R172K (c.515G > A), R172M (c.515G > T) and Pyrosequence for IHD1 R132, IDH2 R172, BRAF V600E, H3F3A K27, H3F3A G34, HIST1H3B, TERT C228T, FGFR1 N546, and FGFR1 K656. The analysis detected a BRAF V600E mutation in both the initial and recurrent tumors, with mutant allele findings at 16 and 49%, respectively. Both 1p/19q and CDKN2A were intact in the initial specimen, but a 19q deletion and CDKN2A homozygous deletion were detected in the recurrent specimen. IDH1/IDH2, H3F3A, HIST1H3B, TERT, and FGFR1 were intact in both specimens. From this analysis the tumor was assumed to have been derived from pleomorphic xanthoastrocytoma.

## Discussion and conclusions

In our case, PXA shifted to a more aggressive phenotype with GBM-like features 12 years after the initial surgery. Despite several reports on malignant transformation from PXA to GBM [2, 8, 9], genetic analyses are absent from most of these cases.

In the current case, the molecular analysis detected BRAF V600E mutations in both the initial and recurrent tumors but the 19q and CDKN2A homozygous deletions observed only in the recurrent specimen served as evidence of malignant transformation. The fact that the tumor origin and BRAFV600E mutations were shared in both resections suggest a PXA-derived pathology but PXA-like histology was absent. Histologically, PXA demonstrates spindle shaped cells, pleomorphic nucleated cells, and EGBs while anaplastic APXA is additionally defined when mitoses number 5 or more per high-power field [1]. These findings were absent in the second specimen and, instead, nuclear atypia, mitotic activity, a diffuse growth pattern, microvascular proliferation and necrosis were observed that led to a histological diagnosis of GBM. In GBM, BRAF V600 E mutations are found in 7.7~9.1% of cases [6, 10] and TERT promoter mutations in 54% of cases [11] while in APXA, 46.2~57% [1, 3], and 23% [11] of cases, respectively (Table 2). From the point of molecular findings, as BRAF V600E mutation was found in 49% and TERT promotor mutations 0% of cells in the second specimen, it was consistent with APXA rather than classic GBM.

**Table 2:** Genetic comparison among the different disease entities

	GBM (wild type)	E-GBM	PXA	APXA	Present case (1st resection)	Present case (2nd resection)
1p/19q	NA	NA	NA	NA	Intact	1p; intact, 19q; deletion
*BRAF V600E*	7.7~9.1% [6, 10]	16.6~93% [11, 13, 14]	63~75% [1, 7]	46.2~57% [1, 3]	Positive (MAF, 16%)	Positive (MAF, 49%)
*CDKN2A/B* homozygous deletion	NA	79% [11]	60~83% [2, 8]	93% [7]	Intact	Positive
*TERT* promoter mutation	54% [11]	71% [11]	4% [11]	23% [11]	Negative	Negative

*GBM* glioblastoma, *E-GBM* epithelioid glioblastoma, *PXA* pleomorphic xanthoastrocytoma, *APXA* anaplastic PXA, *MAF* mutant allele frequency, *NA* not available

Epithelioid GBM (E-GBM), on the other hand, has frequent BRAF V600 E mutations in 16.6~93% of cases [12–14] and E-GBM could arise from PXA, since the BRAF V600 E mutation is shared among these entities [15]. A recent study showed that concurrent BRAF V600E, TERT promoter mutations and CDKN2A/B homozygous deletions were observed in 50% of E-GBM cases [13]. Genetic patterning in our case, including a BRAF V600 E mutation with lack of TERT mutation, was comparable to E-GBM (Table 2); however, no histopathological findings consistent with E-GBM were observed. Although molecular findings showed overlap with E-GBM, neither resection had a histopathology definitive for diagnosis.

There have been several reports of BRAFV600E-inhibitors as a potential treatment option for gliomas [16–19]. Burger et al. described three patients with recurrent malignant gliomas harboring BRAF V600E mutations in which a complete or nearly complete response to dabrafenib was observed after refractoriness to radiotherapy and alkylating chemotherapy [16]. In cases of acquired resistance to BRAF inhibitors, Nicholas et al. describe two cases of successful treatment with dabrafenib and a MEK inhibitor (trametinib) in relapsed APXA after single agent dabrafenib [17]. Therefore, molecular findings are an important consideration in treatment planning within the context of malignant transformation from PXA even if the histological diagnosis is GBM.

In conclusions, we present a case of malignant transformation of PXA into GBM. Although diagnosed as GBM by histology, the molecular findings implied a PXA origin. In order to properly categorize these disease entities, more integrated criteria, including molecular information, may be needed.

## Data Availability

The datasets used and/or analyzed during the current study are available from the corresponding authors on reasonable request.

## Abbreviations

APXA: Anaplastic pleomorphic xanthoastrocytoma
EGB: Eosinophilic granular body
E-GBM: Epithelioid glioblastoma
GBM: Glioblastoma
H&E: Hematoxylin and eosin (staining)
HPF: High-power field
IHC: Immunohistochemical (staining)
MAF: Mutant allele frequency
MVP: Microvascular proliferation
NA: Not available
PDD: Photodynamic diagnosis
PXA: Pleomorphic xanthoastrocytoma
WHO: World Health Organization
